# The effects of road traffic noise on mental performance

**DOI:** 10.1186/1735-2746-10-18

**Published:** 2013-02-09

**Authors:** Iraj Alimohammadi, Raziyeh Soltani, Stephan Sandrock, Manouchehr Azkhosh, Mahmood Reza Gohari

**Affiliations:** 1 Occupational Health Research Center, Tehran University of Medical Sciences, Tehran, Iran; 2 Institut Für Angewandte Arbeitswissenschaft, Dusseldorf, Germany; 3 Faculty of Psycology, University of Social Welfare and Rehabitation Sciences, Tehran, Iran; 4 Department of Biostatistics, Hospital Management Research Center, Tehran university of Medical Sciences, Tehran, Iran

**Keywords:** Traffic noise, Performance, Extra/introversion

## Abstract

**Background:**

Noise is one of the more widespread pollutions of road transportation system, which can cause deterioration in performance. This experimental study was designed to assess the effect of road traffic noise on performance with regard to extra/introversion and sex of participants. The personality trait of extra/introversion has been remarked as relevant factor to mental performance.

**Results:**

Thirty six (26 males and 10 females) medical sciences students of Tehran University participated in the study. The students were placed in an unechoing room and performed the Cognitrone test from Vienna Test System in quiet condition and under road traffic noise (71 dBA). The results of this study pointed out that noise increased the percentage of sum of correct answers but had no effect on the speed of performance. Furthermore this study showed that performance was enhanced in extroverts (P=0.001) but no significant difference was found in introverts (P ≤0.05).

**Conclusions:**

The regression analysis revealed that extra/introversion was more important than sex to predict the performance parameters.

## Background

Prominently and progressively more exposure to road traffic noise is a feature of urban environment. An increasing number of vehicles, difficulty of control of emitted noise and high numbers of exposed persons to noise intensify undesirable transportation noise effects. In the European Union 40% of the inhabitants are exposed to equivalent noise levels exceeding 55 dBA in daytime and more than 30% in nighttime [1]. The equivalent noise level in downtown of Great Tehran was reported 74.7 as dBA [2]. At the present time, noise is more widespread pollution of road transportation system which may cause interference with communications, effects on cardio-vascular system, productivity, social behavior, hearing impairment, disorder in concentration and vigilance, sleep disturbances and noise annoyance [3-5]. It was approved that noisy environment can disturb brain activity, processing of mental tasks, and also my cause tribulation in conversation. Common noise related problems are interference with communication and sleep disturbance [6]. Decreased quality in sleep is considered to be a major health outcome of environmental noise [7]. Noise exposure can also cause other non-auditory effects such as annoyance, changes of behavior and deterioration in performance [8].

Although there are controversial results, but in many studies noise exposure has found to decrease the performance [5]. Non-homogeneity results are partly due to participant individual differences in different experiments. It is experimentally well-known that persons react in different ways to noise. Some of them disturb severely, and performance is increased in the others [9]. Personality variables are one of three groups of factors which influence mental performance in noisy environment [10].

The personality trait of extra/introversion has been remarked as a relevant factor to mental performance [11]. Number of correct responses, reaction time, and some symptoms of exposure to noise such as headache, fatigue and lack of concentration may be considered as performance indices [12,13]. It seems also that noise characteristics and type of task affects level of increasing or decreasing in performance. For example noise has less effect on psychomotor task in comparison with cognitive processes [14].

Generally, it could be said that the effects of noise on performance depends on extra/introversion, neuroticism, and noise sensitivity, although the results in this issue are not homogenous [9]. The level of tolerance to noise in extroverts and introverts is different because of diversity of motivation threshold level. It was found that most desirable noise level in extroverts was higher than in introverts [15,16]. The previous researches have shown that performance of introverts in complex cognitive tasks, in comparison to extroverts, was more affected by interventional factors such as music and background noise [9]. On the other hand, it has been shown that extroverts in presence of noise work faster than introverts [17]. Furthermore, some researchers have shown that the performance of exposed persons to noise could be influenced under different variables such as nature of the task, length of exposure, and sex. Hambrick-dixon [18] showed that only girl’s performance manifested adverse effects of exposure to subway noise over time. The effect of transportation noise on cognitive performance is established. It is shown that road traffic noise has highly significant correlation to cognitive abilities such as working memory and sustained attention [19].

Since the number of exposed persons to traffic noise is increasing and regarding to effects of road traffic noise on performance, this study was conducted. This experimental study was designed to examine the influence of personality trait of intro/extraversion and sex on performance under quiet condition and road traffic noise.

## Methods

Traffic noise was measured at octave band frequency at 6 points in zone no.5 in Tehran for 3 hours between 8 am to 12 am by B and K sound level meter Model 2238. The microphone of sound level meter was located at 120cm in height with 2 meters distance from the edge of the road [20]. The noise measurement points were selected as far away as possible from reflector surfaces, such as buildings, to reduce the reverberant noise. Traffic noise was recorded simultaneously with noise measurement.

The study was conducted on 36 medical sciences students (26 males and 10 females) in Tehran University of Medical Sciences. The students were placed in an acoustical room and performed the Cognitrone test from Vienna Test System under quiet condition two times. The first time was considered as training and its data were not measured. The recorded traffic noise was emitted after 15 minutes at 71 dBA for 2 hours. Then Cognitrone test was performed after noise exposure.

Cognitrone test is used for assessment of attention and concentration through the comparison of figures concerning their congruence. This test is based on Reulecke’s theory [21] in which concentration is considered as state defined by the three variables of energy, performance and accuracy [22]. In this study, test form of S5 was used. Four figures were displayed at top and one figure was displayed under them on a monitor which had 12 inches diameter, 1024*768 resolution, and 69 Hz frequency. If one of four figures was identical with the one of the bottom, subjects had to press a green button and if the figures were not the same a red bottom no action was required. S5 is a limited working time form, in which is the figures disappear after 1.8s if the subject had no response and the next figure appears automatically. Totally this test consists of 200 sets of figures. The variables of sum of correct answers and the mean of working time were considered as performance parameters of the test. Test’s reliability is equal to 0.95 (r = 0.95) [22].

Personality trait of extro/introversion was measured with the Eysenck Personality Inventory [23] that comprises 57 items with a binary scale (yes and no). There are 24 items for extroversion, 24 items for neuroticism and 9 items for subject’s sincerity in answering. Each item was assigned 0 or 1 point in extroversion, neuroticism and lying scales. If the sum of points were more than 12 in extroversion, the subjects are considered as extrovert. No students were omitted in basis of sincerity in answering.

## Results and discussion

Participant’s age ranged from 19 to 35 (mean = 26.33 ± 3.56, S.D = 3.56). The average of equivalent of sound pressure level at five points was 71 dBA. The difference between percentage of sum of correct answer in silence and when exposed to traffic noise was significant (Table 1). Traffic noise enhanced the sum of correct answer in comparison with under quiet. The equivalent noise level under quite condition was 15 dBA. On the other hand the mean of working time of Cognitrone test under quiet and in exposed to traffic noise had not significant difference (Table 1).

**Table 1:** Means and standard deviations of performance parameters in participants

**Performance parameter**	**Mean ± S.D.**	**P value**
Percentage of sum of correct answers under quiet	68 ± 19.80	0.001**
Percentage of sum of correct answers under traffic noise	77.20 ± 15.80	
The mean of working time under quiet	56.30 ± 25.00	0.181
The mean of working time under traffic noise	61.90 ± 21.60	

* significant level at P < 0.01.

Nonparametric Tests, Two- Related-Samples Tests, Wilcoxon.

Statistical analysis also showed that traffic noise improved the correct answers of extroverts and males, but working time of Cognitrone test of participants was not influenced by noise (Tables 2 and 3). The comparison of performance of introverts and extroverts shows that traffic noise increased the percentage of sum correct answers of extroverts. On the other hand no significant differences were found between working time of participants’ test under quiet and traffic noise.

**Table 2:** Comparison between mental performance parameters in extra/introverts under different acoustic conditions

**Extroverts**		**Introverts**		
P value	Mean ± S.D	P value	Mean ± S.D	Performance parameter
0.001**	37.20 ± 9	0.069	31.85 ± 10.86	Percentage of sum of correct answers under quiet
	42.15 ± 6.30		34.86 ± 10.08	Percentage of sum of correct answer under traffic noise
0.191	29.33 ± 10.05	0.460	28.05 ± 14.25	The mean of working time under quiet
	34.15 ± 11.08		30.15 ± 12.66	The mean of working time under traffic noise

*significant level: P < 0/05.

Paired Samples T Test.

**Table 3:** Comparison between mental performance parameters in order to sex under different acoustic conditions

**Male**		**Female**		
P value	Mean ± S.D	P value	Mean ± S.D	Performance parameter
0.001**	35.39 ± 8.55	0.059	34.75 ± 10.63	Percentage of sum of correct answers under quiet
	39.86 ± 7.78		39.15 ± 10.70	Percentage of sum of correct answer under traffic noise
0.078	29.06 ± 12.25	0.867	31.07 ± 13.85	The mean of working time under quiet
	33.34 ± 11.45		31.64 ± 12.55	The mean of working time under traffic noise

*significancy level: P < 0.05.

Paired Samples T Test.

To determine if the increasing in sum correct answers of participants was relevant to extraversion or sex (male) the distribution of sex by intro/extroversion was tested (Table 4). The crosstabulation of sex by extroversion is shown the participants in order to sex were distributed uniformly by intro/extraversion (P _value_= 0.362). The regression analysis revealed that intro/extraversion (standardized coefficient of Beta = −0.358) was a more important factor rather than sex (standardized coefficient of Beta = −0.059) to predict the percentage of sum of correct answer under traffic noise (Table 5).

**Table 4:** Crosstabulation of sex by intro/extraversion

		**Personality**		
		**Introvert**	**Extravert**	**Total**
Sex	Male	10 (38%)	16 (62%)	26 (100%)
	Female	4 (40%)	6 (60%)	10 (100%)

Pearson Chi-Square = 0.829, P _value_ = 0.362.

**Table 5:** Regression models of performance parameters in order to intro/extraversion and sex

	
Percentage of sum of correct answers under quiet = −9.66* extraversion −0.29*sex + 73.57	F = 1.398 (P _value_ = 0.258)
Percentage of sum of correct answer under traffic noise = −11.64* extraversion −1.94*sex + 83.21	F = 3.343 (P _value_ = 0.045^*^)
The mean of working time under quiet = −6.73* extraversion + 5.26*sex + 58.76	F = 0.453 (P _value_ = 0.639)
The mean of working time under traffic noise = −7.72* extraversion −5.07*sex + 67.95	F = 0.849 (P _value_ = 0.435)

*Significant at P < 0.05.

Although the results of researches about relation between noise and performance are mixed but generally noise degrades the performance [14,24]. The results of this study pointed out that the percentage of correct answers under traffic noise was rather more than under quiet. In other words noise increased the accuracy of the subjects. This finding can be explained by arousal theory. Arousability which represents activity level of Central Nervous System (CNS) adjusts human response to stimulus [25]. According to Broadbent [26], noise causes to increase in arousal level by narrowing of attention. This reduction of attention level restricts the range of information processed and led to impair the performance. According to this theory low and high arousal (or low and high level of stress) causes decrement of performance [14]. Regarding to be high the correct answers (performance) of participants under traffic noise one could say the arousal level of subjects was moderate. In other words, if we used the lower or higher equivalent of noise level it might the performance be decrement. It seems the impairment in performance was observed after exposure to between 90 to 100 dB of noise [14]. It has been shown that noise has no effects on the speed of performance, which is conformed to our result [27] (Table 1).

This study shows that extroverts had better performance (sum of correct answers) under noise condition as compared to introverted subjects (Table 2) which this is in accordance with the findings of Belojevic [11]. This finding is also congruent with Eysenck’s proposition that introverts are not able to tolerate stimulation stimuli as extroverts are able to. Introverts and extroverts are different in brain arousal level that is low level of motivation is required for introverts. Because of high level of arousal in introverts, they refuse motivation. Therefore introverts react more intense than extroverts to motivation [9]. It has been shown that preferred noise levels for extroverts are higher than for introverts [16].

Our study revealed that traffic noise increased the accuracy in males, but no effects on female subjects (Table 3). The result of some researches confirmed this finding. Gulian and Thomas [10] studied the effects of gender on arithmetic performance and found that noise clearly reduced the pace at which female subjects were working, but it hardly affected the pace of male subjects. The difference between the effect of noise on performance in male and female may be due to noise sensitivity. Alimohammadi [28] found that the noise sensitivity in females were rather higher than males’. Meanwhile the regression analysis revealed that extra/introversion had more influence to improve of accuracy than gender.

In our experimental study extraverted subjects and males showed better performance in traffic noise than introverts and females respectively. Extraverts and males had better accuracy under noisy condition, but the speed of performance had not varied in noisy condition as compared with quiet condition for extra/introversion and gender factors. Based on the results of this study it could be said that extra/introversion is an important factor that must be regarded for working in noisy environment.

## Conclusion

There are many factors affecting mental performance of exposed persons to noise and therefore the study results are not consistent or are at times even controversial. Both the noise characteristics and the type of tasks, probably influence performance level. This study showed that road traffic noise improves the attention and concentration rather than quiet condition. This finding is probably relevant to enhancing the arousibility level of the participants. The authors suggest to conduct more researches on this issue.

## Competing interests

The authors declare that they have no competing interests.

## Authors’ contribution

All authors read and approved the final manuscript.
